# Tertiary lymphoid organs in wild boar exposed to a low-virulent isolate of African swine fever virus

**DOI:** 10.1080/01652176.2024.2331525

**Published:** 2024-03-27

**Authors:** Néstor Porras, José M. Sánchez-Vizcaíno, Antonio Rodríguez-Bertos, Aleksandra Kosowska, José Á. Barasona

**Affiliations:** a VISAVET Health Surveillance Centre, Complutense University of Madrid, Madrid, Spain; b Department of Animal Health, Faculty of Veterinary, Complutense University of Madrid, Madrid, Spain; c Department of Internal Medicine and Animal Surgery, Faculty of Veterinary, Complutense University of Madrid, Madrid, Spain

**Keywords:** African swine fever virus (ASFV), high-virulent isolate, low-virulent isolate, tertiary lymphoid organs (TLOs), local immunity, wild boar, histopathology, immunohistochemistry

## Abstract

Despite the great interest in the development of a vaccine against African swine fever (ASF) in wild boar, the immunological mechanisms that induce animal protection are still unknown. For this purpose, tertiary lymphoid organs (TLOs) of wild boar were characterised and compared with mucosa-associated lymphoid tissues (MALTs) by histopathology, histomorphometry and immunohistochemistry (CD3, CD79, PAX5, LYVE1, fibronectin). In addition, real-time polymerase chain reaction (qPCR) and immunohistochemistry (p72) were used to evaluate the presence of ASF virus (ASFV) in blood and tissues samples, respectively. TLOs were observed in animals infected with a low-virulent ASFV isolate (LVI), animals co-infected with low and high-virulent ASFV isolates (LVI-HVI) and animals infected only with the high virulence isolate (HVI). TLOs in LVI and LVI-HVI groups were located adjacent to the mucosa and presented a similar structure to MALT. Immunoexpresion of p72 observed in the inflammatory cells adjacent to TLOs/MALTs confirmed its development and reactivity generated by ASF attenuated isolates. Immunohistochemical evaluation, based on cellular composition (T and B lymphocytes), and histomorphometrical study revealed a more pronounced maturation of TLOs/MALTs in the LVI-HVI group. It is currently unclear whether these formations play a protective role by contributing to local immunity in chronic inflammatory diseases. However, the structural similarities between TLOs and MALTs and the location of TLOs close to the mucosa suggest that they may perform a similar function, facilitating a local protective response. Nevertheless, further investigations are warranted to assess the cellular and humoral dynamics of these lymphoid organs induced by attenuated isolates.

## 1. Introduction

African swine fever (ASF) is a haemorrhagic disease affecting domestic pigs and wild boar (*Sus scrofa*) that must be notified to the World Organisation for Animal Health (WOAH) (WOAH 2023). The causative agent of ASF is a large, enveloped, double-stranded DNA virus, which is the only member of the genus Asfivirus within the family Asfarviridae (Arias et al. 2002).

ASF is currently a serious socioeconomic and health-related threat to the global swine industry (Sánchez-Cordón et al. 2018). Europe is currently subjected to an ongoing emergency aggravated by the lack of a commercial vaccine and effective treatments against ASF (Sánchez-Vizcaíno et al. 2015; WAHIS: World Animal Health Information System 2023). Moreover, wild boar plays a key role in both the spread of the infection and its maintenance within the neighbouring countries of the European Union (EU) (Chenais et al. 2018). This is due to the increase in wild boar population density, multiple entry points of the disease through infected wild boar, the location of farms and its poor biosecurity measures (Cadenas-Fernández et al. 2019; Jurado et al. 2018). The behaviour of wild boars, such as their scavenging, is also very important since ASFV is mainly transmitted by contact with blood (Barasona et al. 2021; Cukor et al. 2019). However, there are very few published studies on the histopathological lesions found in the different forms of disease in wild boars infected with ASFV isolates of different virulence (Pietschmann et al. 2015; Pikalo et al. 2020; Rodríguez-Bertos et al. 2020; Sánchez-Cordón et al. 2019; Sehl et al. 2020). Despite the highly problematic role of wild boar in the spread and maintenance of the disease, current studies on the development of an ASFV vaccine are mainly focused on domestic pigs (Barasona et al. 2019).

The development of a vaccine has been additionally hampered by ASFV genetic complexity, gaps in the knowledge concerning ASFV infection and immunity, the lack of development of neutralizing antibodies in animals and the lack of stable cell lines for research, among others (Arias et al. 2018; Barasona et al. 2019; Blome et al. 2020). Therefore, our research team has conducted experimental studies to evaluate the safety and efficacy of naturally attenuated isolates of ASFV as vaccine candidates for wild boar, demonstrating its potential to induce an immune response against highly virulent isolates (Barasona et al. 2019; Rodríguez-Bertos et al. 2020).

On the other hand, the subsequent histopathological evaluation of ASFV-infected animals (from the previous experiments discussed above) revealed a possible new immunological mechanism related to chronic inflammation. This finding was characterised by the formation of tertiary lymphoid organs (TLOs) in multiple locations. It is well known that lymphoid neogenesis can be induced during chronic inflammatory processes, such as infection and autoimmunity, leading to the formation of germinal centres and ectopic T-cell areas (Aloisi and Pujol-Borrell 2006). Moreover, previous studies have suggested that TLOs formed in infected tissues probably play a protective role and function to contain local infection (Aloisi and Pujol-Borrell 2006; Carragher et al. 2008).

This work aims to characterise and compare the formation of TLOs with mucosa-associated lymphoid tissues (MALT) present in ASFV-infected wild boar by histological, histomorphometric and immunohistochemical analyses. In addition, we also studied the relationship between the structure and reactivity of TLOs and the presence of ASFV isolates of different virulence. These studies could be an important starting point as regards determining the function of these TLOs in the immune protection systems against highly virulent isolates.

## 2. Material and methods

### 2.1. Experimental design

The experimental design and sample collection used in this study were based on previous animal experiments described in Barasona et al. (2019) and Rodríguez-Bertos et al. (2020). All experiments performed in this study were approved by the Ethics Committee of the Community of Madrid (reference PROEX 124/18), and were carried out under biosafety level 3 (BSL-3) conditions at the VISAVET Centre of the Complutense University of Madrid (UCM) in accordance with European, national and regional regulations.

Thirty-eight female wild boar piglets, aged 3-4 months and weighing 10-15 kg, were obtained from a commercial wild boar farm, which had tested negative for the following main porcine pathogens in the region: Aujeszky virus, *Mycobacterium bovis, Mycoplasma hyopneumoniae* and type 2 porcine circovirus.

**Figure 1.**
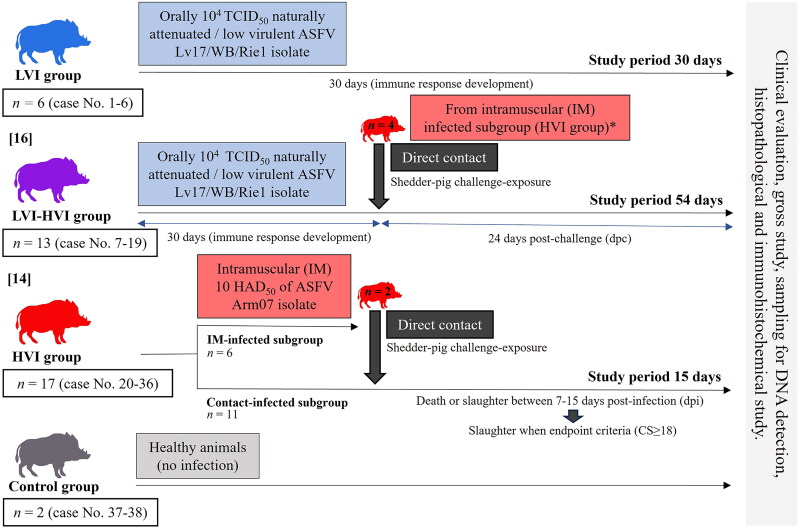
Scheme of the study design, including the four groups (LVI, LVI-HVI, HVI and the control group), from prime inoculation to the end of the experiment (15, 30, 54 days post-infection).

Four different groups were included in this study: low-virulent ASFV isolate (LVI) group, low and high-virulent ASFV isolates (LVI-HVI) group, high-virulent ASFV isolate (HVI) group and the negative control group (Figure 1). The first group (LVI group) consisted of six animals orally infected with 10^4^ TCID_50_ of the low virulence isolate Lv17/WB/Rie1 of naturally attenuated ASF virus, with demonstrated potential to induce an immune response in both domestic pigs and wild boar (Barasona et al. 2019; Gallardo et al. 2019). The second group (LVI-HVI group) was composed of 13 animals orally infected with 10^4^ TCID_50_ of the attenuated ASFV Lv17/WB/Rie1 isolate and exposed to a highly virulent isolate (Armenia07 (Arm07)). Both groups (LVI and LVI-HVI) had the same immunization period (30 days post-infection (dpi)). However, animals in the LVI group were sacrificed at 30 dpi, while animals in the LVI-HVI group were sacrificed 24 days later after challenge with the highly virulent isolate in order to evaluate the protection conferred by this isolate. The third group (HVI group) consisted of 17 animals, six of which were infected intramuscularly with a highly virulent isolate (Arm07) (excretory animals) and the remaining 11 animals by exposure to the excretory animal group. Animals within HVI group were kept up to a limit of 15 dpi. In addition, four of the six excretory animals (*n* = 4) within HVI group were used to challenge LVI-HVI animals. Finally, the fourth group (negative control group) was composed of two healthy animals not infected by any ASFV isolate.

### 2.2. Clinical evaluation

Animals were observed daily throughout the trial in order to monitor their health status. This was done using a videocamera (recording 24 h a day) and by means of visits from a veterinarian specialising in wildlife. The evolution of the ASFV infection was evaluated in terms of a quantitative clinical score (CS) in accordance with specific parameters for ASFV infection in wild boar described by Cadenas-Fernández et al. (2020). This CS includes rectal temperature, behaviour, body condition, alterations in skin, ocular/nasal discharge, the swelling of joints, respiratory symptoms, digestive symptoms, and neurological symptoms. Fever was defined as a rectal temperature above 40.0 °C. All clinical observations were recorded on a daily basis, with the exception of temperature in order to minimise animal handling and stress. The humane endpoint was pre-defined as animals with a CS > 18, and animals that showed severe clinical signs (level 4) of fever, behaviour, body condition, respiratory and digestive symptoms for more than two consecutive days were also included, following the guidelines described by Cadenas-Fernández et al. (2020). Any animals that were, according to veterinary criteria, suffering unacceptably without reaching the pre-defined humane endpoint were also euthanised.

### 2.3. Sampling

EDTA-blood was collected from each animal once a week during the study period. Viral DNA was extracted from 200 µl of each sample using the High Pure Template Preparation Mix Kit (Roche Diagnostics GmbH, Mannheim, Germany) according to the manufacturer’s instructions. A post-mortem examination was performed on all the animals at the end of the study. A total of 20 tissue samples were collected from each animal, including lymph nodes (mandibular, renal, mediastinal, retropharyngeal, mesenteric, prescapular, gastrohepatic, inguinal lymph nodes), spleen, liver, lung, heart, kidney, brain, urinary bladder, intestine, diaphragm, bone marrow and synovial membranes. All tissues were taken in order to assess histopathological changes, immunohistochemical analysis and ASFV DNA detection (qPCR).

### 2.4. Pathological study

During the necropsies, macroscopic lesions were evaluated following a previously performed protocol (Rodríguez-Bertos et al. 2020) based on the study by Galindo-Cardiel et al. (2013). In the current study, successive cuts were made during trimming to obtain three cassettes of the same tissue. The three cassettes of the 20 tissue samples collected from each animal were fixed in 10% neutral formalin for 72 h, automatically processed (Citadel 2000 Tissue Processor, Thermo Fisher Scientific, Waltham, MA), and embedded in paraffin (HistoStar Embedding Workstation, Thermo Fisher Scientific). Consecutive sections of four µm thickness were obtained for each cassette using a microtome (FinesseMe+, Thermo Fisher Scientific), and were stained with hematoxylin-eosin (Gemini AS Automated Slide Stainer, Thermo Fisher Scientific). In all cases in which TLO/MALT were observed, ten consecutive four µm thick sections were subsequently made for each cassette using a microtome.

### 2.5. Immunohistochemical examination

The paraffin sections placed on positively charged glass slides were deparaffinised in xylene and rehydrated. This step was carried out using the Epredia PT module Deparaffin and Heat Induced Epitope Retrieval (HIER). Endogenous peroxidase was blocked by immersing the samples in 3% hydrogen peroxide in a methanol solution (PanreacQuímica S.L.U.) for 15 min. The samples were then incubated with 2.5% normal horse serum (ImmPRESS ® VR Horse antimouse IGG Polymer Kit, Vector Laboratories) for blocking (RTU) for 1 h. The slides were subsequently incubated overnight at 4 °C with the primary antibodies detailed in Table 1 (DAKO, Glostrup, Denmark; Thermo Fisher Scientific, Waltham, MA, USA; Abcam, Cambridge, UK; Ingenasa, Madrid, Spain). Positive and negative controls were included in each batch of slides. For negative controls, the primary antibody was omitted and substituted for tris-buffered saline. A spleen from a wild boar infected with the Arm07 ASFV isolate was used as a positive control. The secondary antibody (ImmPRESS ® VR Horse AntiMouse/AntiRabbit IGG Polymer Kit, Peroxidase; Vector Laboratories) was added the following morning and incubated for 1 h. The revealing process was carried out using peroxidase (ImmPACT ® NovaRED®Substrate Kit Peroxidase) and the samples were then counter-stained with hematoxylin (Gemini AS Automated Slide Stainer, Thermo Fisher Scientific).

**Table 1.** List of antibodies used in the immunohistochemistry study.

Antibody	Type	Host	Dilution	Company
Anti-CD3	Polyclonal	Rabbit	1:100	DAKO
Anti-PAX5	Monoclonal	Mouse	Ready to use	DAKO
Anti-CD79	Monoclonal	Mouse	1:50	DAKO
Anti-LYVE1	Polyclonal	Rabbit	1:100	Thermo Fisher Scientific
Anti-Fibronectin	Polyclonal	Rabbit	1:100	Abcam
Anti-VP72 (18BG3) ASF	Monoclonal	Mouse	1:100	Ingenasa

The histopathological and immunohistochemical evaluation for the detection and characterisation of TLOs was carried out according to previously proposed criteria (Hiraoka et al. 2016; Neyt et al. 2012) based on cellular composition and vascular structure. CD3 (T-cells) and CD79/PAX(B-cells) were, therefore, used to characterise the phenotype lymphocytes, while LYVE1 was used for the lymphatic vessels and fibronectin for the reticullar extracellular matrix of which the microchannels (conduits) are composed. The presence of ASFV near TLOs was detected by performing a semi-quantitative assessment of cells immunolabeled for ASF viral antigen (protein p72-18BG3) on different organs, which were evaluated as follows: immunolabeled mononuclear cells were counted in 5 adjacent (close to the lymphoid formations) non-overlapping fields under high-power field (HPF) magnification (400×). A score from 0 to 4 was assigned to each sample: (0) no presence of immunolabeled cells; (1) 1–10 immunolabeled cells; (2) >10-25 immunolabeled cells; (3) >25-100 immunolabeled cells; (4) >100 immunolabeled cells.

### 2.6. Histomorphometric study

Ten consecutive sections of the three cassettes in each organ were evaluated under a light microscope in order to locate all TLO/MALT-compatible formations and their morphometric changes. The histomorphometric evaluation was performed using an image analyser (Leica Application Suite v.4, Leica Wetzlar, Germany) and the total length and width of each lymphoid formation were measured by means of CD3 immunohistochemistry. Moreover, an immunohistochemical study of PAX5 was employed in order to obtain the length and width of the germinal centre.

### 2.7. Real-time polymerase chain reaction (qPCR)

A total of 20 tissue samples were collected from each animal. DNA extraction was performed immediately after sample collection. Positive qPCR results were determined by identifying the threshold cycle value (Ct) at which reporter dye emission appeared above background fluorescence within 40 cycles. For this study, only the blood ASFV Ct values obtained on the last day (day of euthanasia) were considered. The detection of the ASFV DNA in blood and tissue samples was performed using the Universal Probe Library (UPL) real-time PCR (qPCR) recommended by the World Organisation for Animal Health (WOAH) and previously described by Fernández-Pinero et al. (2013). Positive and negative controls were used in DNA extraction and qPCR. The positive control employed was a well-known and sequenced sample of the virulent ASFV isolate, while the negative control was nuclease-free sterile water.

### 2.8. Statistical analysis

TLOs and MALTs are three-dimensional formations and the histomorphometric study cannot consider their total depth. Therefore, to reduce this limitation and obtain more reliable results, this methodology analysed changes in the width and length of ten tissue sections per cassette made at different depths (30 sections total). Then the average of all histomorphometric measurements obtained from each lymphoid formation was calculated. Differences in the occurrence of TLOs and MALTs among groups were assessed using a Pearson’s Chi-square test, while differences in the histomorphometric results (total size, germinal centre size, width and length averages) of TLOs/MALTs among groups were assessed using one-way ANOVA test. All statistical analyses were performed in R Core Team (2023).

## 3. Results

### 3.1. Clinical evaluation and gross pathological findings

Animals in the LVI group showed no relevant clinical signs and only one animal presented transient fever. Necropsy findings showed mild gross lesions, mainly mild hyperaemic splenomegaly accompanied by white pulp hypertrophy (6/6), slight hepatomegaly with congestion (3/6) and occasional and minimal subpleural multifocal petechial haemorrhages in the lung (5/6).

In the LVI-HVI group, no relevant clinical signs were observed, except for fever during viraemia. The post-mortem analysis revealed no pathological findings compatible with ASF, only mild macroscopic lesions similar to those found in the LVI group.

Clinical signs of animals in the HVI group were characterised by increased body temperature, decreased alertness, walking difficulties, generalised erythema, slight ocular discharge and digestive symptoms such as mucus in the stools and sporadic vomiting. These animals died between 7 and 15 days post-infection (dpi). In addition, the main necropsy findings comprised moderate to severe ascites, hydrothorax and hydropericardium. Pulmonary oedema, congestion and multifocal haemorrhages on the lung surface were observed. There was also congestion, splenomegaly, hepatomegaly, lymphadenomegaly and presence of haemorrhages of variable severity in the lymph nodes, kidney, urinary bladder and intestinal mucosa. Animals in the HVI group died between 7 and 15 days post-infection (dpi).

No relevant clinical signs or gross lesions were observed in the negative control group.

### 3.2. Histopathological, histomorphometrical and immunohistochemical evaluation

The formation of immune cell aggregates, composed of T and B lymphocytes, accompanied by blood and lymphatic vessels and resembling a secondary lymphoid organ (SLO) structure was observed in 15/36 cases (42%). Depending on the location of these lymphoid aggregates, it was possible to differentiate between tertiary lymphoid organs (TLOs) and secondary lymphoid organs (SLOs), specifically mucosa-associated lymphoid tissues (MALTs).

MALT observed in wild boars included gut-associated lymphoid tissues (GALTs) located (in solitary form) in the duodenum and (clustered together) in the jejunum, and bronchus-associated lymphoid tissues (BALTs), which were located perivascularly and were not close to the bronchus or bronchiole. In addition, there were infrequent isolated localisations in the gallbladder, urinary bladder and oesophagus (Table 2). Furthermore, the TLOs found in this study were mainly located in the kidney, but also in the synovial membrane and pancreas.

**Table 2.** ASFV Ct values, distribution of lymphoid tissues and p72-ASF immunoexpression score for each animal.

Group	Case n°	ASFV Ct values (blood)	MALT/TLOs location	p72-ASF immunoexpression*
LVI	1	39.5	Oesophagus/kidney	1/2
	2	Negative	–	–
	3	31	Duodenum	2/1
	4	28	Gallbladder	2
	5	32	Kidney/urinary bladder	2/0
	6	35	Oesophagus/kidney/duodenum	1/2/2
LVI-HVI	7	Negative	Kidney/duodenum	2/3
	8	Negative	–	–
	9	Negative	Kidney	3
	10	Negative	Kidney	2
	11	Negative	Lung	2
	12	Negative	Synovial membrane	2
	13	Negative	Oesophagus/kidney	2/1
	14	Negative	Oesophagus/kidney	2/2
	15	Negative	Oesophagus	2
	16	Negative	Kidney	2
	17	Negative	–	–
	18	Negative	–	–
	19	Negative	–	–
HVI	20	30.6	–	–
	21	16.6	Pancreas/duodenum	2/3
	22	22	–	–
	23	22.2	–	–
	24	16.7	–	–
	25	14.7	–	–
	26	24.6	–	–
	27	16.4	–	–
	28	17.1	–	–
	29	15.5	–	–
	30	16.6	–	–
	31	24.1	–	–
	32	17.2	–	–
	33	19.9	–	–
	34	18.2	–	–
	35	18.6	–	–
	36	19.1	–	–
Control	37	Negative	–	–
	38	Negative	–	–

* Immunohistochemical score (p72 ASF): 0 (negative); 1 (minimal); 2 (mild); 3 (moderate); 4 (severe).

**Figure 2.**
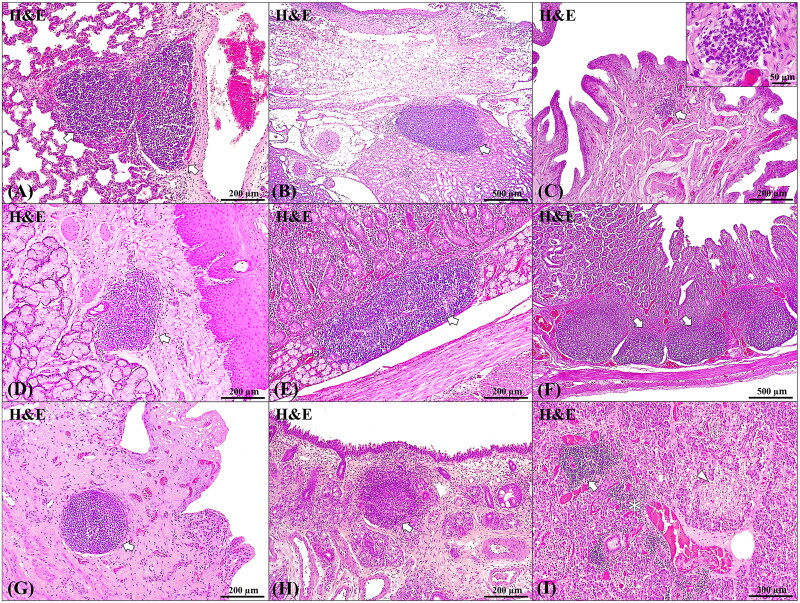
Histopathological description of lymphoid aggregate formations at several locations observed in ASFV-infected wild boar. Lymphoid aggregates (arrows) in lung (A), kidney (B), urinary bladder (C), oesophagus (D), duodenum (E), jejunum (F), gallbladder (G), synovial membrane (H) and pancreas (I). (A) Round to ovoid perivascular lymphoid structure in the lung interstitium (arrows), covered with epithelium. H&E stain, 100x. (B) Ovoid perivascular lymphoid structure in the medullar interstitium of the renal hilum, near the epithelium of the renal calyx, covered with epithelium. H&E stain, 40x. (C) Irregularly shaped perivascular lymphoid structure in the lamina propria of the urinary bladder (arrow). H&E stain, 100x. Inset, 400x. (D) Ovoid perivascular lymphoid structure in the mucous gland of the oesophagus (arrow), covered with epithelium. H&E stain, 100x. (E) Ovoid perivascular lymphoid structure in the submucosa, between brunner’s glands (arrow), covered with epithelium. H&E stain, 100x. (F) Round to ovoid perivascular lymphoid structure in the submucosa (arrows). H&E stain, 40x. (G) Round perivascular lymphoid structure in lamina propria of the gallbladder (arrow), covered with epithelium. HE stain, 100x. (H) Round perivascular lymphoid structure in the sublining layer in the synovial membrane (arrow). H&E stain, 100x. (I) Irregularly shaped perivascular lymphoid structure in the pancreas, close to inflammatory foci (asterisk) and necrotic acinar cells (arrowhead). H&E stain, 100x.

Lymphoid organs were found in five animals from the LVI group (83.3%) and were observed in several locations, mainly the kidney (renal hilum) (3/6). They were also found associated with the mucosa in the lamina propria of the urinary bladder, (1/6), gallbladder (1/6), and oesophagus (2/6), (with the latter located between the submucosal glands). In addition, lymphoid aggregates corresponding to GALTs were also present, but in less frequent locations such as the duodenum (2/6). In the LVI-HVI group, 9/13 animals (69.2%) had these lymphoid formations, located mainly in the kidney (6/13), but also in the oesophagus (3/13), duodenum (2/13), lung (1/13) (perivascularly) and synovial membrane (in the connective tissue of the sublining layer) (1/13). Only 1/17 animal (6%) from the HVI group had these lymphoid formations in two organs: the pancreas and the duodenum (1/17). No such lymphoid structures were observed in any organs of the animals in the control group. Figure 2 shows images of the lymphoid formations at the locations observed in this study.

The presence of these lymphoid formations was statistically significant in all the groups compared to the control group (*p* < 0.005), which did not show formations compatible with TLOs, despite the fact that the HVI group only reported one TLO case. However, no significant differences were demonstrated between LVI and LVI-HVI groups.

**Figure 3.**
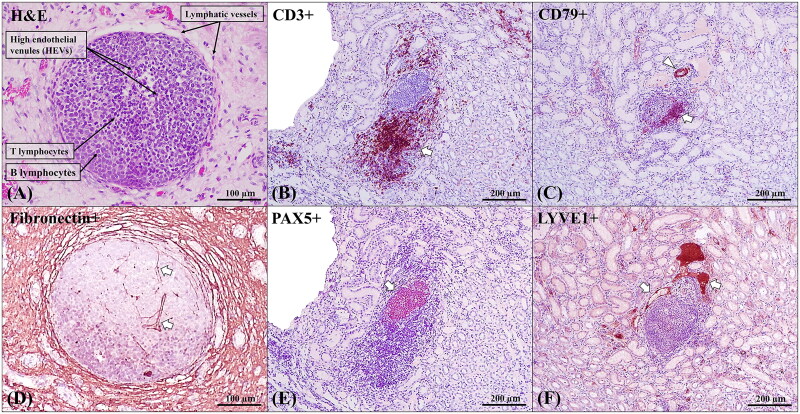
Histopathological and immunohistochemical characterisation of the TLOs in ASF infected tissues of wild boar under experimental conditions. (A) Evaluation of histological structure and cell composition. The TLOs were composed of B and T lymphocytes, with high endothelial venules (HEVs) on the inside, and lymphatic vessels surrounding the formation. HE stain, 200x. (B) T-cells (arrow) have marked CD3^+^ immunoexpression. Rabbit polyclonal anti-CD3, 100x. (C) B-cells (arrow) and blood vessels (arrowhead) have marked CD79^+^ immunoexpression. Mouse monoclonal rabbit anti-CD79, 100x. (D) Marked reticular extracellular matrix fibronectin^+^ immunoexpression inside the TLO (arrow), compatible with formation of microchannels (conduits). Rabbit polyclonal anti-fibronectin; 100x. (E) B-cells (arrow) have marked PAX5^+^ immunoexpression. Mouse monoclonal rabbit anti-PAX5; 100x. (F) Lymphatic vessels (arrow) have marked LYVE1^+^ immunoexpression. Rabbit polyclonal anti-LYVE1, 100x.

In this work, these structures were detected and characterised by means of an immunohistochemical study carried out in accordance with previously proposed criteria (Hiraoka et al. 2016; Neyt et al. 2012) (Figure 3). T lymphocytes were detected by employing CD3^+^ immune-labelling, and B lymphocytes were detected through the use of PAX-5^+^ and CD79^+^ immune-labelling. In addition, anti-CD79 antibody stained blood vessels as a result of immunoreactivity against B lymphocytes recruited at that level and, secondarily, against endothelial cells. Lymphatic vessels were detected by employing LYVE1^+^ immune-labelling. Furthermore, immuno-labelling was used to detect fibronectin in the reticular extracellular matrix from which microchannels (ducts) are formed. These lymphoid formations also presented different degrees of development and cell maturation.

**Figure 4.**
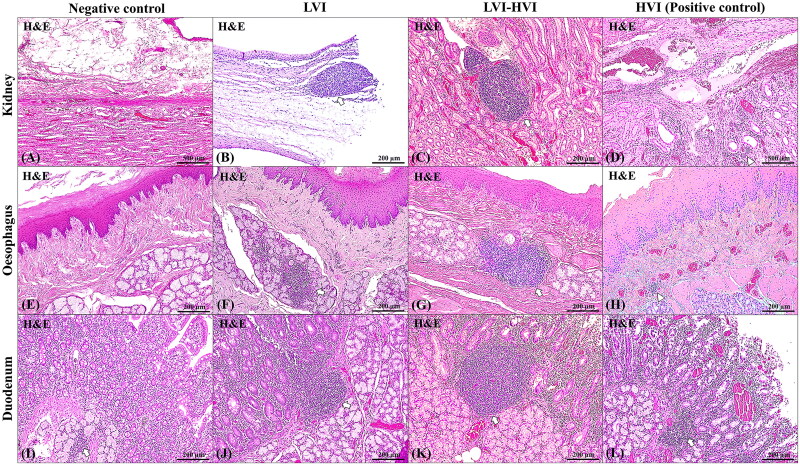
Comparative histopathological TLO/MALT evaluation comparing negative control (A,E,I), LVI (B,F,J), LVI-HVI (C,G,K) and HVI (D,H,L) groups as regards kidney (A,B,C,D), oesophagus (E,F,G,H) and duodenum (I,J,K,L). Negative control animals had neither lesions nor lymphoid aggregations in kidney; H&E stain, 40x (A). Negative control animals had neither lesions nor lymphoid aggregations in oesophagus (E), only a reminiscence composed of T lymphocytes in the duodenum (arrow) (I); H&E stain, 100x. LVI and LVI-HVI groups had well-demarcated and developed lymphoid formations (arrows) (B,F,J,C,G,K), which were larger and more organised in shape and structure in the LVI-HVI group (C,G,K); H&E stain, 100x. HVI (positive control) group had lesions typical of acute ASF form (D,H,L); H&E stain, 40x. In the renal pelvis, severe vasodilation, congestion and perivascular haemorrhages, along with multifocal interstitial pyelonephritis (arrowhead) were observed; H&E stain, 40x (D). These vascular alterations were also present in the mucosa of oesophagus and duodenum (H,L), with perivascular mononuclear inflammation in oesophagus (arrowhead) and diffuse mononuclear inflammation in lamina propria of duodenum H&E stain, 40x. T-lymphocyte aggregation was observed only in the duodenal submucosa (arrow) H&E stain, 40x (L).

**Figure 5.**
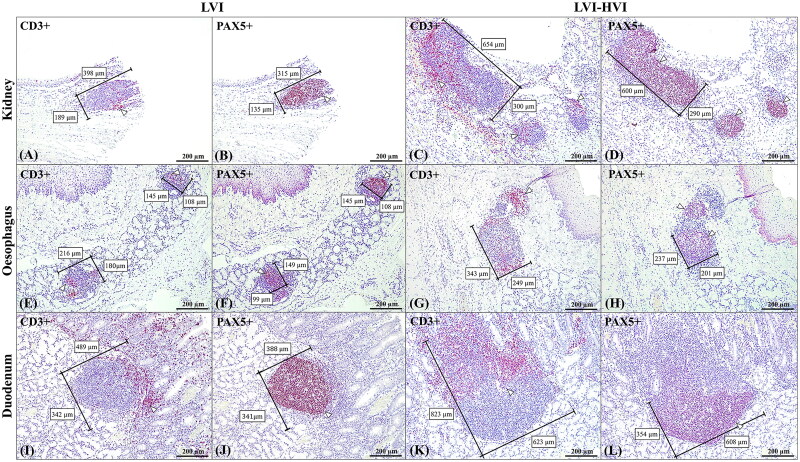
Histomorphometrical and immunohistochemical (CD3-PAX5) TLO/MALT evaluation comparing LVI (A,B,E,F,I,J) and LVI-HVI (C,D,G,H,K,L) groups as regards kidney (A,B,C,D), oesophagus (E,F,G,H), duodenum (I,J,K,L). Total length and width were measured histomorphometrically by means of CD3 immunohistochemistry; 100x (A,C,E,G,I,K). Germinal centre length and width were obtained using PAX5 immunohistochemistry; 100x (B,D,F,H,J,L). Higher histomorphometric values were observed in the LVI-HVI group. Brown immunolabelling is indicated by arrowheads.

Differences between LVI and LVI-HVI groups with regard to the development and maturation of ­lymphoid structures were assessed by means of histopathological, histomorphological and immunohistochemical analysis (Figures 3, 4). Thus, a trend in the increase in TLO/MALT size was observed in the LVI-HVI group with respect to the LVI group, with statistically significant differences in the enlargement of the width (*p* < 0.05). Most of the cases in the LVI group showed isolated lymphoid aggregations, with an average size of 321.7 × 202.2 (length = 321.7 µm, 95% confidence interval (CI): 266.9-376.4; width =202.2 µm, 95% CI: 173.4-231). Those aggregations were immature and poorly demarcated, presenting a diffuse zone of CD3^+^ T-cells and a variable number of PAX-5^+^ and CD79^+^ B-cells sometimes arranged in a moderate delimited germinal centre, with an average size of 271.9 × 162.8 µm (length =271.9 µm, 95% CI: 205.4-338.3; width =162.8 µm, 95% CI: 131.4-194.2). Animals from the LVI-HVI group had more numerous, organised and well-demarcated lymphoid structures, with an average size of 404 × 271.1 µm (length =404 µm, 95% CI: 351.5-456.5; width =271.1 µm, 95% CI: 234.4-307.7), a clearly delineated CD3^+^ T-cell zone and a larger and more organised PAX-5^+^ and CD79^+^ germinal centre with an average size of 287.6 × 223.9 µm (length =287.6 µm, 95% CI: 248.6-326.5; width =223.9 µm, 95% CI: 195-252.8) (see Figures 4, 5). See Supplementary Table 1 for more detailed information on the histomorphometric results.

**Figure 6.**
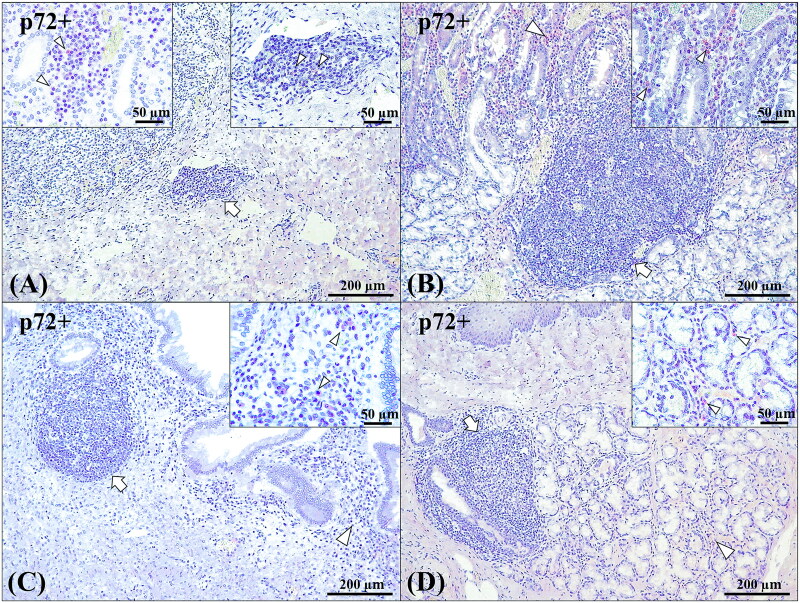
Anti-p72 ASF immunoexpression in TLOs/SLOs or neighbouring areas. (A) Lymphoid aggregation in the renal calyx (arrow); 100x. Inset (left): p72^+^ cells inside the TLO (arrowheads); 40x. Inset (right): p72^+^ cells in the peritubular interstitium medulla (arrowheads); 400x. (B) Lymphoid aggregation in the duodenum (arrow); 100x. Inset: p72^+^ cells in the lamina propria (arrowheads); 400x. (C) Lymphoid aggregation in the lamina propria of the gallbladder (arrow); 100x. Inset: p72^+^ cells in the lamina propria (arrowheads); 400x. (D) Lymphoid aggregation in the mucous glands of the oesophagus (arrow); 100x. Inset: p72^+^ cells between the mucous glands (arrowheads); 400x. Brown immunolabelling is indicated by arrowheads.

The presence of ASFV and its possible relationship with TLO formation and the development and reactivity of MALT was evaluated by performing an immunohistochemical study against p72 of the ASFV, linked to a score system (Table 2). The results obtained confirmed the presence of the virus in all the tissues studied, with the exception of TLOs in the urinary bladder (Case No. 5). P72^+^ cells were mainly observed in the periphery of the lymphoid tissue, with higher immunoexpression in cases in which there was an associated inflammatory infiltrate. Very few positive cells were observed in those cases in which there was no longer an associated chronic inflammatory infiltrate. However, it was possible to observe p72^+^ cells within the TLO in some cases from the LVI group, mainly in the kidney, where the TLO was still at an early stage of formation (Figure 6).

### 3.3. Viral loads in blood and tissue samples

The LVI group had low blood viral loads at 30 dpi (Ct = 34.3 ± 5.1), although two animals exhibited maximum Ct values (Ct ≥ 39) at the time of sacrifice. Furthermore, all tissue samples from one of the six wild boars in the LVI group tested positive for ASFV DNA detection (Ct = 33.39 ± 2.02), while the remaining animals had positive qPCR results (Ct = 34.9 ± 0.9) for an average of four tissues, mainly involving the submandibular and retropharyngeal lymph nodes.

The LVI-HVI group had sporadic peaks of viraemia during the prime inoculation and co-infection with the virulent isolate (Ct = 39.00 ± 0.78), although a negative result was obtained for all the animals at 54 dpi (24 dpc). ASFV genomic DNA was not detected in any tissue from five of the animals in this group, while the remaining animals showed weakly positive qPCR results (Ct = 38.41 ± 1.16) for an average of five tissues (Barasona et al. 2019). Moreover, a large proportion (80%) of animal tissues from LVI and LVI-HVI groups that developed TLOs were negative for ASFV by qPCR at the time of sacrifice.

Animals in the HVI group had high blood viral loads (Ct = 25.18 ± 7.37). In addition, all animal tissues were positive for ASFV DNA detection by qPCR (Ct = 23.46 ± 1.58) (Rodríguez-Bertos et al. 2020).

The control group did not have viraemia and no ASFV DNA was detected in animal tissues (Table 2).

## 4. Discussion

There are still many gaps in the understanding of the immune response induced by ASFV infection. This study has determined a possible new immunological mechanism in chronic ASFV infection, characterised by the formation of tertiary lymphoid organs (TLOs) that have not been previously described in any histopathological study concerning ASF disease, regardless of the virulence of the ASFV isolate.

It is first necessary to differentiate between the cases of TLOs and SLOs. In that of TLOs, some locations have already been observed in previous studies, mainly in humans but also in pigs. These locations include the kidney (Pezzolato et al. 2012; Robson and Kitching 2020), which has been observed more frequently, and also the synovial membrane (Manzo et al. 2010) and the pancreas (Hiraoka et al. 2016; Tschernig et al. 2016). With regard to SLOs, although mucosa-associated lymphoid tissues (MALTs) have been physiologically observed in pigs (Jørgensen et al. 2022; Sarradell et al. 2003; Stefanov et al. 2021; Urmila et al. 2019), some locations such as the oesophagus, gallbladder and urinary bladder are not as frequent.

TLOs have been described/characterised in humans according to the criteria proposed by Neyt et al. (2012). The histopathological and immunohistochemical findings of this study meet the previous established criteria for TLOs regarding the presence of B-cell follicles, T-cell zones, blood and lymphatic vessels (Neyt et al. 2012). TLOs share cell structural homology with SLOs, but TLOs are not encapsulated and supplied by afferent lymphatic vessels (Hiraoka et al. 2016). Furthermore, these TLOs were observed at different stages of development, as also occurred in previous studies, in which three evolutionary stages were defined according to the appearance of the different structures that characterise the identification of these formations (Luo et al. 2019). This has also been observed in our study, in which significant differences between groups were detected thanks to the exhaustive immunohistochemical and histomorphometric study of these lymphoid structures. The LVI-HVI group showed a greater number and size of these lymphoid aggregations, which were more mature and had a more developed germinal centre due to longer antigenic exposure and the chronicity of the disease. This reveals a process of lymphoid activation against the virus, leading to the development of antibodies with a local function.

Previous studies in humans have shown that TLO neogenesis develops in areas of immune stimulation with local pathology resulting from chronic infection or inflammation (Aloisi and Pujol-Borrell 2006; Carragher et al. 2008; Manzo et al. 2010; Yin et al. 2017). Previous studies have also demonstrated an association between the development of TLOs and the severity of organ injury or inflammation (Manzo et al. 2010; Robson and Kitching 2020; Sato et al. 2020). Although different infectious or inflammatory agents cause the same degree of chronic inflammation and inflict similar lesions, their ability to produce TLO formation can vary widely (Neyt et al. 2012). There is increasing evidence that TLOs represent an adaptation of the body to an increased demand for a local immune response (Carragher et al. 2008; Neyt et al. 2012). There are several diseases and experimental models in which TLOs have been found in both mice and humans, including microbial agents, autoimmune disease, chronic transplant rejection, degenerative/environmental causes, idiopathic disorders, and cancer (Yin et al. 2017). These formations were found after humans and mice became infected with certain viral agents such as influenza, HIV, vaccinia virus Ankara and γ-herpesvirus-68 in the lungs, and hepatitis C virus in the liver (Carragher et al. 2008; Luo et al. 2019; Neyt et al. 2012). In our study, TLOs were observed in several of the organs of ASFV-infected wild boars and were most frequently detected in the kidney.

The presence of ASFV was confirmed by immunohistochemistry, which allowed us to observe p72^+^ cells in almost all lymphoid tissues (TLO/MALT) of the animals in LVI and LVI-HVI groups of this study. This may be explained by the fact that infections with low virulence ASFV isolates cause mild to moderate inflammatory lesions that persist for a sustained period of time, resulting in the formation of such lymphoid structures. Furthermore, the qPCR analysis showed the absence or minimal presence of viral loads in blood and some animal tissues, mainly in LVI and LVI-HVI groups. However, certain lymphoid organs showed positive qPCR and immunohistochemistry (p72+ cells) results in the lymphoid formations studied, indicating the longer persistence of viral loads in certain lymphoid tissues even in the absence of viraemia (Abworo et al. 2017; Ståhl et al. 2019). In an experiment with the influenza virus, it has been observed that dendritic cells remain in the associated lymphoid tissue long after the virus has been cleared for presentation to memory T-cells and therefore it is likely that T-cells capture viral antigens retained from the follicular dendritic cell (FDC) networks (Kim et al. 2010; Muniz et al. 2011). This could explain why TLOs exist in animals in which the viral load has already disappeared. Moreover, subsequent exposure to new more virulent antigens allows the reactivation and maintenance of these lymphoid organs (Muniz et al. 2011).

MALTs function as a tissue barometer responsible for maintaining immune homeostasis and regulating host antimicrobial immunity against invasive pathogens (Jones et al. 2016), thereby enhancing immune tolerance in mucosal compartments. It is, therefore, increasingly recognised that antigen-specific responses can also be generated at sites separate from these SLOs such as TLOs, playing a similar role in the immune response (Jones et al. 2016; Neyt et al. 2012). Although TLOs functions will depend on multiple factors such as their location, the stimulus that generated them, the kinetics of inflammation and the cellular activation produced (Barone et al. 2016; Pabst 2007).

Furthermore, it has been postulated that a distinction should be made between physiological and pathological TLOs and the lymphatic pathway system (Barone et al. 2016). Physiological TLOs are caused by constant antigenic exposure and are usually mucosa-associated, while pathological TLOs are related to autoimmune diseases (Barone et al. 2016). The great number of autoimmune diseases that could involve joints, thyroid, salivary glands, liver or chronic inflammation, such as hepatitis C virus*, Helicobacter pylori*, *Borellia burgdorferi*, and the rejection of several transplanted organs makes it necessary to study in more detail the formation mechanism and the role played by TLOs in the different scenarios (Carragher et al. 2008; Pabst 2007; Shomer et al. 2003).

It is well known that the clinical course and survival time vary greatly depending on the ASFV isolate administered (Sánchez-Cordón et al. 2019; Sánchez-Vizcaíno et al. 2015). In this study, ASFV HVI caused acute disease with a survival time of 7 to 15 dpi and 100% mortality. This mortality is linked to the inoculation route, since animals infected by contact died later (reaching up to 15 dpi); this is due to the incubation period required for intramuscularly injected animals to infect the remaining (contact-infected) animals. Animals infected only with LVI showed minimal chronic disease and no mortality, similar to the LVI-HVI group, which showed a survival rate of 92% (Barasona et al. 2019). These animals were euthanised at 30 dpi (LVI group) and 54 dpi (24 dpc) (LVI-HVI group). It should be noted that animals from LVI and LVI-HVI groups with mucosa-associated TLOs survived and had minimal clinical signs and histopathological findings. However, no such formations were observed in the HVI group, except for Case No. 21 (13 dpi), which developed a TLO in the pancreas that was not associated with the mucosa and had severe adjacent inflammatory and necrotic lesions. These results suggest that there is no direct relationship between TLOs formation and the intensity of the lesions and clinical signs, but rather it has more to do with the chronicity of the process.

There are examples in which ectopic lymphoid tissues appear to contribute to local protective systems that control local immunity by initiating and amplifying the immune response (Carragher et al. 2008; Luo et al. 2019; Neyt et al. 2012). However, there are indications that ectopic follicles may be controlling or imprinting particular characteristics in locally generated immune responses, independently of the activities of conventional lymphoid organs (Carragher et al. 2008). Furthermore, Sánchez-Cordón et al. (2021) suggested that MALTs may be involved in the selective absorption of the ASFV in intranasally inoculated pigs, thereby stimulating the immune response and modulating and controlling virus replication and spread (Sánchez-Cordón et al. 2021). It is therefore possible that, depending on the location of the lymphoid tissues and whether or not they are associated with the mucosa, the immune response may have a beneficial or pathological effect (Pabst 2007). Our results are consistent with those described above, since we observed moderate presence of p72+ cells in areas where reactive MALTs were present (oesophagus, duodenum, jejunum and gallbladder). Similar immunohistochemical findings were observed in cases of TLOs in the kidney, as they also formed in the renal calyx, close to the urothelium. Although the presence of TLOs in the kidney has been reported to be associated with severe inflammatory lesions (Robson and Kitching 2020; Sato et al. 2020), it is possible that it may have a beneficial effect if it is located close to the mucosa, mimicking the function performed by MALTs.

In summary, this study reveals the development and reactivity of TLOs/MALTs generated by ASF attenuated isolates, with a more pronounced maturation of TLOs in cases of reinfection with the highly virulent isolate. This observation is substantiated by the presence of ASFV-p72 within the inflammatory infiltrate adjacent to these lymphoid organs. Furthermore, the structural similarities between TLOs and SLOs, coupled with their proximity to the mucosa, suggest that TLOs may facilitate a local protective response, emulating the role of MALTs. Nevertheless, further investigations are warranted to assess the cellular and humoral dynamics of these lymphoid organs induced by attenuated isolates or, even, whether they may be a valuable option to promote local immunity.

## Supplementary Material

Supplemental Material

## Data Availability

The original contributions presented in the study are included in the paper/supplementary material; further inquiries can be directed to the corresponding author.

## References

[CIT0001] Abworo EO, Onzere C, Oluoch Amimo J, Riitho V, Mwangi W, Davies J, Blome S, Peter Bishop R. 2017. Detection of African swine fever virus in the tissues of asymptomatic pigs in smallholder farming systems along the Kenya-Uganda border: implications for transmission in endemic areas and ASF surveillance in East Africa. J Gen Virol. 98(7):1806–1814. doi: 10.1099/jgv.0.00084. 28721858

[CIT0002] Aloisi F, Pujol-Borrell R. 2006. Lymphoid neogenesis in chronic inflammatory diseases. Nat Rev Immunol. 6(3):205–217. doi: 10.1038/nri1786. 16498451

[CIT0003] Arias M, Jurado C, Gallardo C, Fernández-Pinero J, Sánchez-Vizcaíno JM. 2018. Gaps in African swine fever: analysis and priorities. Transbound Emerg Dis. 65 Suppl 1:235–247. doi: 10.1111/tbed.12695. 28941208

[CIT0004] Arias M, Sánchez-Vizcaíno JM, et al. 2002. African swine fever. In: Morilla A, editor. Trends in emerging viral infections of swine. Ames, Iowa: Iowa State Press; p. 119–124. doi: 10.1002/9780470376812.ch4a.

[CIT0005] Barasona JA, Cadenas-Fernández E, Kosowska A, Barroso-Arévalo S, Rivera B, Sánchez R, Porras N, Gallardo C, Sánchez-Vizcaíno JM. 2021. Safety of African swine fever vaccine candidate Lv17/WB/Rie1 in wild boar: overdose and repeated doses. Front Immunol. 12:761753. doi: 10.3389/fimmu.2021.761753. 34917082 PMC8669561

[CIT0006] Barasona JA, Gallardo C, Cadenas-Fernández E, Jurado C, Rivera B, Rodríguez-Bertos A, Arias M, Sánchez-Vizcaíno JM. 2019. First oral vaccination of eurasian wild boar against african swine fever virus genotype II. Front Vet Sci. 6:137. doi: 10.3389/fvets.2019.00137. 31106218 PMC6498142

[CIT0007] Barone F, Gardner DH, Nayar S, Steinthal N, Buckley CD, Luther SA. 2016. Stromal fibroblasts in tertiary lymphoid structures: a novel target in chronic inflammation. Front Immunol. 7:477. doi: 10.3389/fimmu.2016.00477. 27877173 PMC5100680

[CIT0008] Blome S, Franzke K, Beer M. 2020. African swine fever - A review of current knowledge. Virus Res. 287:198099. doi: 10.1016/j.virusres.2020.198099. 32755631

[CIT0009] Cadenas-Fernández E, Sánchez-Vizcaíno JM, Kosowska A, Rivera B, Mayoral-Alegre F, Rodríguez-Bertos A, Yao J, Bray J, Lokhandwala S, Mwangi W, et al. 2020. Adenovirus-vectored African swine fever virus antigens cocktail is not protective against virulent Arm07 isolate in Eurasian wild boar. Pathogens. 9(3):171. doi: 10.3390/pathogens9030171. 32121082 PMC7157622

[CIT0010] Cadenas-Fernández E, Sánchez-Vizcaíno JM, Pintore A, Denurra D, Cherchi M, Jurado C, Vicente J, Barasona JA. 2019. Free-ranging domestic pig and wild boar interactions in an endemic area of African swine fever. Front Vet Sci. 6:376. doi: 10.3389/fvets.2019.00376. 31737649 PMC6831522

[CIT0011] Carragher DM, Rangel-Moreno J, Randall TD. 2008. Ectopic lymphoid tissues and local immunity. Semin Immunol. 20(1):26–42. doi: 10.1016/j.smim.2007.12.004. 18243731 PMC2276727

[CIT0012] Chenais E, Ståhl K, Guberti V, Depner K. 2018. Identification of wild boar-habitat epidemiologic cycle in african swine fever epizootic. Emerg Infect Dis. 24(4):810–812. doi: 10.3201/eid2404.172127. 29553337 PMC5875284

[CIT0013] Cukor J, Linda R, Václavek P, Mahlerová K, Šatrán P, Havránek F. 2019. Confirmed Cannibalism in wild boar and its possible role in African swine fever transmission. Transbound Emerg Dis. 67(3):1068–1073. doi: 10.1111/tbed.13468. 31886951

[CIT0014] Fernández-Pinero J, Gallardo C, Elizalde M, Robles A, Gómez C, Bishop R, Heath L, Couacy-Hymann E, Fasina FO, Pelayo V, et al. 2013. Molecular diagnosis of African Swine Fever by a new real-time PCR using universal probe library. Transbound Emerg Dis. 60(1):48–58. doi: 10.1111/j.1865-1682.2012.01317. 22394449

[CIT0015] Galindo-Cardiel I, Ballester M, Solanes D, Nofrarías M, López-Soria S, Argilaguet JM, Lacasta A, Accensi F, Rodríguez F, Segalés J. 2013. Standardization of pathological investigations in the framework of experimental ASFV infections. Virus Res. 173(1):180–190. doi: 10.1016/j.virusres.2012.12.018. 23313935

[CIT0016] Gallardo C, Soler A, Rodze I, Nieto R, Cano-Gómez C, Fernandez-Pinero J, Arias M. 2019. Attenuated and non-haemadsorbing (non-HAD) genotype II African swine fever virus (ASFV) isolated in Europe, Latvia 2017. Transbound Emerg Dis. 66(3):1399–1404. doi: 10.1111/tbed.13132. 30667598

[CIT0017] Hiraoka N, Ino Y, Yamazaki-Itoh R. 2016. Tertiary lymphoid organs in cancer tissues. Front Immunol. 7:244. doi: 10.3389/fimmu.2016.00244. 27446075 PMC4916185

[CIT0018] Jones GW, Hill DG, Jones SA. 2016. Understanding immune cells in tertiary lymphoid organ development: it is all starting to come together. Front Immunol. 7:401. doi: 10.3389/fimmu.2016.00401. 27752256 PMC5046062

[CIT0019] Jørgensen PB, Eriksen LL, Fenton TM, Bailey M, Agace WW, Mörbe UM. 2022. The porcine large intestine contains developmentally distinct submucosal lymphoid clusters and mucosal isolated lymphoid follicles. Dev Comp Immunol. 131:104375. doi: 10.1016/j.dci.2022.104375. 35219758

[CIT0020] Jurado C, Fernández-Carrión E, Mur L, Rolesu S, Laddomada A, Sánchez-Vizcaíno JM. 2018. Why is African swine fever still present in Sardinia? Transbound Emerg Dis. 65(2):557–566. doi: 10.1111/tbed.12740. 29027378

[CIT0021] Kim TS, Hufford MM, Sun J, Fu YX, Braciale TJ. 2010. Antigen persistence and the control of local T cell memory by migrant respiratory dendritic cells after acute virus infection. J Exp Med. 207(6):1161–1172. doi: 10.1084/jem.20092017. 20513748 PMC2882836

[CIT0022] Luo S, Zhu R, Yu T, Fan H, Hu Y, Mohanta SK, Hu D. 2019. Chronic inflammation: a common promoter in tertiary lymphoid organ neogenesis. Front Immunol. 10:2938. doi: 10.3389/fimmu.2019.02938. 31921189 PMC6930186

[CIT0023] Manzo A, Bombardieri M, Humby F, Pitzalis C. 2010. Secondary and ectopic lymphoid tissue responses in rheumatoid arthritis: from inflammation to autoimmunity and tissue damage/remodeling. Immunol Rev. 233(1):267–285. doi: 10.1111/j.0105-2896.2009.00861.x. 20193005

[CIT0024] Muniz LR, Pacer ME, Lira SA, Furtado GC. 2011. A critical role for dendritic cells in the formation of lymphatic vessels within tertiary lymphoid structures. J Immunol. 187(2):828–834. doi: 10.4049/jimmunol.1004233. 21666055 PMC3137511

[CIT0025] Neyt K, Perros F, GeurtsvanKessel CH, Hammad H, Lambrecht BN. 2012. Tertiary lymphoid organs in infection and autoimmunity. Trends Immunol. 33(6):297–305. doi: 10.1016/j.it.2012.04.006. 22622061 PMC7106385

[CIT0026] Pabst R. 2007. Plasticity and heterogeneity of lymphoid organs. What are the criteria to call a lymphoid organ primary, secondary or tertiary? Immunol Lett. 112(1):1–8. doi: 10.1016/j.imlet.2007.06.009. 17698207

[CIT0027] Pezzolato M, Maina E, Lonardi S, Bozzetta E, Grassi F, Scanziani E, Radaelli E. 2012. Development of tertiary lymphoid structures in the kidneys of pigs with chronic leptospiral nephritis. Vet Immunol Immunopathol. 145(1–2):546–550. doi: 10.1016/j.vetimm.2011.12.011. 22227076

[CIT0028] Pietschmann J, Guinat C, Beer M, Pronin V, Tauscher K, Petrov A, Keil G, Blome S. 2015. Course and transmission characteristics of oral low-dose infection of domestic pigs and European wild boar with a Caucasian African swine fever virus isolate. Arch Virol. 160(7):1657–1667. doi: 10.1007/s00705-015-2430-2. 25916610

[CIT0029] Pikalo J, Schoder M-E, Sehl J, Breithaupt A, Tignon M, Cay AB, Gager AM, Fischer M, Beer M, Blome S. 2020. The African swine fever virus isolate Belgium 2018/1 shows high virulence in European wild boar. Transbound Emerg Dis. 67(4):1654–1659. doi: 10.1111/tbed.13503. 32009303

[CIT0030] R Core Team. 2023. R: a language and environment for statistical computing [Internet]. Viena: R Foundation for Statistical Computing. https://www.R-project.org/.

[CIT0031] Robson KJ, Kitching AR. 2020. Tertiary lymphoid tissue in kidneys: understanding local immunity and inflammation. Kidney Int. 98(2):280–283. doi: 10.1016/j.kint.2020.04.026. 32709287

[CIT0032] Rodríguez-Bertos A, Cadenas-Fernández E, Rebollada-Merino A, Porras-González N, Mayoral-Alegre FJ, Barreno L, Kosowska A, Tomé-Sánchez I, Barasona JA, Sánchez-Vizcaíno JM. 2020. Clinical course and gross pathological findings in Wild boar infected with a highly virulent isolate of african swine fever virus genotype II. Pathogens. 9(9):688. doi: 10.3390/pathogens9090688. 32842614 PMC7559345

[CIT0033] Sánchez-Cordón PJ, Floyd T, Hicks D, Crooke HR, McCleary S, McCarthy RR, Strong R, Dixon LK, Neimanis A, Wikström-Lassa E, et al. 2021. Evaluation of lesions and viral antigen distribution in domestic pigs inoculated intranasally with African swine fever virus Ken05/Tk1 (Genotype X). Pathogens. 10(6):768. doi: 10.3390/pathogens10060768. 34207265 PMC8234863

[CIT0034] Sánchez-Cordón PJ, Montoya M, Reis AL, Dixon LK. 2018. African swine fever: a re-emerging viral disease threatening the global pig industry. Vet J. 233:41–48. doi: 10.1016/j.tvjl.2017.12.025. 29486878 PMC5844645

[CIT0035] Sánchez-Cordón PJ, Nunez A, Neimanis A, Wikström-Lassa E, Montoya M, Crooke H, Gavier-Widén D. 2019. African swine fever: disease dynamics in wild boar. Experimentally infected with ASFV isolates belonging to genotype I and II. Viruses. 11(9):852. doi: 10.3390/v11090852. 31540341 PMC6783972

[CIT0036] Sánchez-Vizcaíno JM, Mur L, Gomez-Villamandos JC, Carrasco L. 2015. An update on the epidemiology and pathology of African swine fever. J Comp Pathol. 152(1):9–21. doi: 10.1016/j.jcpa.2014.09.003. 25443146

[CIT0037] Sarradell J, Andrada M, Ramírez AS, Fernández A, Gómez-Villamandos JC, Jover A, Lorenzo H, Herráez P, Rodríguez F. 2003. A morphologic and immunohistochemical study of the bronchus-associated lymphoid tissue of pigs naturally infected with Mycoplasma hyopneumoniae. Vet Pathol. 40(4):395–404. doi: 10.1354/vp.40-4-395. 12824511

[CIT0038] Sato Y, Boor P, Fukuma S, Klinkhammer BM, Haga H, Ogawa O, Floege J, Yanagita M. 2020. Developmental stages of tertiary lymphoid tissue reflect local injury and inflammation in mouse and human kidneys. Kidney Int. 98(2):448–463. doi: 10.1016/j.kint.2020.02.023. 32473779

[CIT0039] Sehl J, Pikalo J, Schäfer A, Franzke K, Pannhorst K, Elnagar A, Blohm U, Blome S, Breithaupt A. 2020. Comparative Pathology of domestic pigs and wild boar infected with the moderately virulent African swine fever virus isolate “estonia 2014. Pathogens. 9(8):662. doi: 10.3390/pathogens9080662. 32824331 PMC7459997

[CIT0040] Shomer NH, Fox JG, Juedes AE, Ruddle NH. 2003. Helicobacter-induced chronic active lymphoid aggregates have characteristics of tertiary lymphoid tissue. Infect Immun. 71(6):3572–3577. doi: 10.1128/IAI.71.6.3572-3577.2003. 12761142 PMC155770

[CIT0041] Ståhl K, Sternberg-Lewerin S, Blome S, Viltrop A, Penrith ML, Chenais E. 2019. Lack of evidence for long term carriers of African swine fever virus - a systematic review. Virus Res. 272:197725. doi: 10.1016/j.virusres.2019.197725. 31430503

[CIT0042] Stefanov I, Stefanov S, Tsandev N, et al. 2021. Morphometric characteristics of the lymphatic nodules in the porcine gallbladder. Acta Morphologica et Anthropologica. 28:3–4.

[CIT0043] Tschernig T, Neuner F, Albrecht AS, Lang I, Meier C, Jörns A, Pabst R. 2016. Tertiary lymphoid tissue occurs in the porcine pancreas. Pancreas. 45(3):e6–7. doi: 10.1097/MPA.0000000000000554. 26863379

[CIT0044] Urmila TS, Ramayya PJ, Lakshmi MS, Kumar AVNS. 2019. Histomorphological studies on gut associated lymphoid tissue of pig (Sus scrofa). Int J Chem Stud. 8(1):01–03. doi: 10.22271/chemi.2020.v8.i1a.8295.

[CIT0045] WAHIS: World Animal Health Information System. 2023. [accessed July 26]. https://wahis.woah.org/#/home.

[CIT0046] WOAH. 2023. Terrestrial animal health code. Terrestrial code online access; [accessed March 14]. https://www.woah.org/en/what-we-do/standards/codes-and-manuals/terrestrial-code-online-access/.

[CIT0047] Yin C, Mohanta S, Maffia P, Habenicht AJR. 2017. Editorial: tertiary Lymphoid Organs (TLOs): Powerhouses of disease immunity. Front Immunol. 8:228. doi: 10.3389/fimmu.2017.00228. 28321222 PMC5337484

