# Supplementary material for: Tertiary lymphoid organs in wild boar exposed to a low-virulent isolate of African swine fever virus
Source: Vet Q. 2024 Mar 27;44(1):1–13. doi: 10.1080/01652176.2024.2331525 (PMC10976996; doi:10.1080/01652176.2024.2331525)
Supplement: Supplemental Material [file TVEQ_A_2331525_SM0008.docx]

**Supplementary Table 1.** Comparative histomorphometrical analysis (µm) of the lymphoid formations observed in the four groups (negative, LVI, LVI-HVI, HVI).

| **HISTOMORPHOMETRICAL ANALYSIS (µm)*** | | | | | | | | | | | | | | | | |
| --- | --- | --- | --- | --- | --- | --- | --- | --- | --- | --- | --- | --- | --- | --- | --- | --- |
| **ORGAN** | **Negative control** | | | | **LVI** | | | | **LVI-HVI** | | | | **HVI** | | | |
|  | **TOTAL** | | **GERMINAL CENTER** | | **TOTAL** | | **GERMINAL CENTER** | | **TOTAL** | | **GERMINAL CENTER** | | **TOTAL** | | **GERMINAL CENTER** | |
|  | **Length** | **Width** | **Length** | **Width** | **Length** | **Width** | **Length** | **Width** | **Length** | **Width** | **Length** | **Width** | **Length** | **Width** | **Length** | **Width** |
| **Kidney** | **-** | **-** | **-** | **-** | 307 | 158 | 237 | 107 | 451 | 258 | 216 | 197 | **-** | **-** | **-** | **-** |
|  | **-** | **-** | **-** | **-** | 289 | 201 | 209 | 155 | 301 | 298 | 268 | 231 | **-** | **-** | **-** | **-** |
|  | **-** | **-** | **-** | **-** | 401 | 155 | 388 | 140 | 401 | 301 | 395 | 231 | **-** | **-** | **-** | **-** |
|  | **-** | **-** | **-** | **-** | 311 | 169 | 207 | 160 | 344 | 187 | 223 | 186 | **-** | **-** | **-** | **-** |
|  | **-** | **-** | **-** | **-** | 365 | 174 | 198 | 132 | 365 | 210 | 198 | 132 | **-** | **-** | **-** | **-** |
|  | **-** | **-** | **-** | **-** | 409 | 201 | 355 | 135 | 290 | 270 | 249 | 230 | **-** | **-** | **-** | **-** |
|  | **-** | **-** | **-** | **-** | 899 | 403 | 731 | 310 | 210 | 158 | 190 | 158 | **-** | **-** | **-** | **-** |
|  | **-** | **-** | **-** | **-** | 475 | 246 | 450 | 195 | 384 | 260 | 332 | 260 | **-** | **-** | **-** | **-** |
|  | **-** | **-** | **-** | **-** | 278 | 106 | 201 | 80 | 661 | 300 | 600 | 290 | **-** | **-** | **-** | **-** |
|  | **-** | **-** | **-** | **-** | 290 | 109 | 211 | 89 | 188 | 187 | 189 | 187 | **-** | **-** | **-** | **-** |
|  | **-** | **-** | **-** | **-** | 249 | 101 | **-** | **-** | 155 | 140 | 160 | 140 | **-** | **-** | **-** | **-** |
|  | **-** | **-** | **-** | **-** | 123 | 65 | **-** | **-** | 664 | 212 | 371 | 212 | **-** | **-** | **-** | **-** |
|  | **-** | **-** | **-** | **-** | 355 | 269 | 315 | 221 | 178 | 200 | 230 | 200 | **-** | **-** | **-** | **-** |
|  | **-** | **-** | **-** | **-** | **-** | **-** | **-** | **-** | 395 | 124 | 300 | 120 | **-** | **-** | **-** | **-** |
|  | **-** | **-** | **-** | **-** | **-** | **-** | **-** | **-** | 264 | 175 | 261 | 175 | **-** | **-** | **-** | **-** |
|  | **-** | **-** | **-** | **-** | **-** | **-** | **-** | **-** | 359 | 260 | 156 | 140 | **-** | **-** | **-** | **-** |
|  | **-** | **-** | **-** | **-** | **-** | **-** | **-** | **-** | 503 | 394 | 280 | 245 | **-** | **-** | **-** | **-** |
|  | **-** | **-** | **-** | **-** | **-** | **-** | **-** | **-** | 460 | 171 | 466 | 124 | **-** | **-** | **-** | **-** |
|  | **-** | **-** | **-** | **-** | **-** | **-** | **-** | **-** | 570 | 292 | 295 | 182 | **-** | **-** | **-** | **-** |
|  | **-** | **-** | **-** | **-** | **-** | **-** | **-** | **-** | 1059 | 493 | 855 | 450 | **-** | **-** | **-** | **-** |
|  | **-** | **-** | **-** | **-** | **-** | **-** | **-** | **-** | 296 | 193 | 270 | 180 | **-** | **-** | **-** | **-** |
|  | **-** | **-** | **-** | **-** | **-** | **-** | **-** | **-** | 406 | 290 | 310 | 233 | **-** | **-** | **-** | **-** |
|  | **-** | **-** | **-** | **-** | **-** | **-** | **-** | **-** | 523 | 321 | 351 | 301 | **-** | **-** | **-** | **-** |
|  | **-** | **-** | **-** | **-** | **-** | **-** | **-** | **-** | 248 | 154 | 211 | 143 | **-** | **-** | **-** | **-** |
| **Oesophagus** | **-** | **-** | **-** | **-** | 221 | 188 | 99 | 149 | 250 | 225 | 130 | 120 | **-** | **-** | **-** | **-** |
|  | **-** | **-** | **-** | **-** | 151 | 139 | 108 | 145 | 248 | 343 | 201 | 237 | **-** | **-** | **-** | **-** |
|  | **-** | **-** | **-** | **-** | 126 | 132 | 121 | 90 | 461 | 408 | 252 | 227 | **-** | **-** | **-** | **-** |
|  | **-** | **-** | **-** | **-** | 292 | 230 | 156 | 111 | 612 | 334 | 345 | 230 | **-** | **-** | **-** | **-** |
|  | **-** | **-** | **-** | **-** | 380 | 282 | 150 | 178 | 447 | 239 | 274 | 221 | **-** | **-** | **-** | **-** |
|  | **-** | **-** | **-** | **-** | 272 | 216 | 260 | 165 | 160 | 115 | 160 | 110 | **-** | **-** | **-** | **-** |
|  | **-** | **-** | **-** | **-** | 290 | 220 | **-** | **-** | 173 | 86 | 168 | 83 | **-** | **-** | **-** | **-** |
|  | **-** | **-** | **-** | **-** | 301 | 251 | **-** | **-** | 281 | 206 | 278 | 206 | **-** | **-** | **-** | **-** |
|  | **-** | **-** | **-** | **-** | 322 | 193 | **-** | **-** | 611 | 499 | 203 | 183 | **-** | **-** | **-** | **-** |
|  | **-** | **-** | **-** | **-** | **-** | **-** | **-** | **-** | 355 | 312 | 267 | 233 | **-** | **-** | **-** | **-** |
|  | **-** | **-** | **-** | **-** | **-** | **-** | **-** | **-** | 246 | 202 | 150 | 183 | **-** | **-** | **-** | **-** |
|  | **-** | **-** | **-** | **-** | **-** | **-** | **-** | **-** | 410 | 278 | 326 | 241 | **-** | **-** | **-** | **-** |
|  | **-** | **-** | **-** | **-** | **-** | **-** | **-** | **-** | 588 | 280 | 233 | 260 | **-** | **-** | **-** | **-** |
| **Duodenum** | **-** | **-** | **-** | **-** | 482 | 338 | 396 | 374 | 624 | 823 | 354 | 608 | 161 | 66 | **-** | **-** |
|  | **-** | **-** | **-** | **-** | 346 | 332 | 223 | 178 | 479 | 301 | 378 | 290 | **-** | **-** | **-** | **-** |
|  | **-** | **-** | **-** | **-** | **-** | **-** | **-** | **-** | **-** | **-** | **-** | **-** | **-** | **-** | **-** | **-** |
|  | **-** | **-** | **-** | **-** | **-** | **-** | **-** | **-** | **-** | **-** | **-** | **-** | **-** | **-** | **-** | **-** |
|  | **-** | **-** | **-** | **-** | 638 | 299 | 422 | 142 | 462 | 437 | 440 | 421 | **-** | **-** | **-** | **-** |
| **Gallbladder** | **-** | **-** | **-** | **-** | 346 | 319 | **-** | **-** | **-** | **-** | **-** | **-** | **-** | **-** | **-** | **-** |
| **Lung** | **-** | **-** | **-** | **-** | 268 | 172 | **-** | **-** | 601 | 258 | 301 | 425 | **-** | **-** | **-** | **-** |
|  | **-** | **-** | **-** | **-** | 244 | 192 | **-** | **-** | 371 | 320 | 295 | 254 | **-** | **-** | **-** | **-** |
|  | **-** | **-** | **-** | **-** | 301 | 216 | **-** | **-** | 620 | 314 | 510 | 312 | **-** | **-** | **-** | **-** |
|  | **-** | **-** | **-** | **-** | **-** | **-** | **-** | **-** | **-** | **-** | **-** | **-** | **-** | **-** | **-** | **-** |
| **Urinary bladder** | **-** | **-** | **-** | **-** | 129 | 105 | **-** | **-** | **-** | **-** | **-** | **-** | **-** | **-** | **-** | **-** |
|  | **-** | **-** | **-** | **-** | 112 | 87 | **-** | **-** | **-** | **-** | **-** | **-** | **-** | **-** | **-** | **-** |
| **Pancreas** | **-** | **-** | **-** | **-** | **-** | **-** | **-** | **-** | **-** | **-** | **-** | **-** | 256 | 154 | **-** | **-** |
|  | **-** | **-** | **-** | **-** | **-** | **-** | **-** | **-** | **-** | **-** | **-** | **-** | 201 | 171 | **-** | **-** |
| **Synovial membrane** | **-** | **-** | **-** | **-** | **-** | **-** | **-** | **-** | 262 | 194 | 157 | 149 | **-** | **-** | **-** | **-** |
|  | **-** | **-** | **-** | **-** | **-** | **-** | **-** | **-** | 245 | 176 | 143 | 135 | **-** | **-** | **-** | **-** |
| **TOTAL AVERAGE** | - | - | - | - | 321,7 | 202,2 | 271,9 | 162,8 | 404,0 | 271,1 | 287,6 | 223,9 | 206,0 | 130,3 | - | - |

* Each result included in the table is the average obtained from 10 histomorphological measurements of the same lymphoid formation (from the 10 consecutive sections).
